# 2-Dodecyl-6-Methoxycyclohexa-2,5-Diene-1,4-Dione Ameliorates Diabetic Cognitive Impairment Through Inhibiting Hif3α and Apoptosis

**DOI:** 10.3389/fphar.2021.708141

**Published:** 2021-12-16

**Authors:** Lihui Wang, Jinjin Cao, Qianqian Xu, Xiaomei Lu, Xin Yang, Qiong Song, Shuai Chen, Kechen Du, Renbin Huang, Chunlin Zou

**Affiliations:** ^1^ Key Laboratory of Longevity and Aging-Related Diseases of Chinese Ministry of Education, Center for Translational Medicine and School of Preclinical Medicine, Guangxi Medical University, Nanning, China; ^2^ Department of Pharmacology, Guangxi Medical University, Nanning, China

**Keywords:** DMDD, diabetic cognitive impairment, db/db mice, neuronal apoptosis, Hif3a

## Abstract

Diabetes mellitus (DM) is an independent risk factor for cognitive impairment. Although the etiology of diabetic cognitive impairment is complex and multifactorial, the hippocampus neuronal apoptosis is recognized as a main cause of diabetes-induced cognitive impairment. 2-Dodecyl-6-methoxycyclohexa-2,5-diene-1,4-dione (DMDD) was purified from the roots of *Averrhoa carambola* L. Previous research demonstrated that DMDD was safe and effective in delaying some diabetic complications. However, the efficacy of DMDD to ameliorate diabetic cognitive impairment in type 2 diabetes mice has not been reported. In the present study, the behavioral evaluation was performed by Y maze and novel object recognition in db/db mice. Gene expression profiles were detected using mouse lncRNA microarray analysis in the hippocampi of db/db mice. Changes in the neurodegeneration-associated proteins and the apoptosis-related proteins were determined in both db/db mice and high glucose-treated HT22 cells by Western blotting. We observed that DMDD treatment significantly ameliorated the spatial working memory and object recognition memory impairment in db/db mice. Further study showed that neurodegeneration-associated protein tau was decreased after DMDD treatment in the hippocampi of db/db mice. Eleven lncRNAs and four mRNAs including pro-apoptotic gene Hif3a were significantly differently expressed after DMDD treatment in the hippocampi of db/db mice. The expression of Hif3a, cleaved parp, and caspase 3 proteins was significantly increased in the hippocampi of diabetic db/db mice compared with db/m control mice and then decreased after DMDD treatment. Similar beneficial effects of DMDD were observed in HG-treated HT22 cells. These data indicate that DMDD can alleviate cognitive impairment by inhibiting neuronal apoptosis through decreasing the expression of pro-apoptotic protein Hif3a. In conclusion, our study suggests that DMDD has great potential to be a new preventive and therapeutic compound for diabetic cognitive impairment.

## Introduction

Diabetes mellitus (DM) is a complex metabolic disturbances characterized by chronic hyperglycemia. Epidemiological findings indicate that DM is a strong and independent risk factor for cognitive impairment (Chatterjee et al., 2016), Parkinson’s disease (PD) (Athauda and Foltynie, 2016), and Alzheimer’s disease (AD) (Zilliox et al., 2016). The pathophysiology of cognitive dysfunction in diabetes is multifactorial, including insulin signaling defect, inflammatory pathways, oxidative stress, mitochondrial abnormalities, and Tau signaling (Zilliox et al., 2016; Bharadwaj et al., 2017). Cognitive deficits can occur in the early stages of diabetes, which can be exacerbated by metabolic syndrome (Yaffe et al., 2004). With the prolongation of life span, the need for new methods for preventing the development of neurodegenerative disease in T2DM is becoming more and more urgent.

The link between diabetes and neurodegenerative disease has been well established, but the underlying mechanisms of pathological development remains unclear. Previous studies focused on tauopathies, α-synuclein accumulation (Ashraf et al., 2014), mitochondrial dysfunction, and apoptosis (Cheng et al., 2019). Multiple studies showed that diabetes-related metabolic dysfunction affected tau phosphorylation and resulted in hyperphosphorylated tau accumulates to form neurofibrillary tangles (NFTs) and lead to neuronal death (Zhang et al., 2018; Ho et al., 2020). In addition, α-synuclein accumulation plays a key role in diabetic cognitive impairment. Under pathological conditions, misfolded α-synuclein aggregates into a form of high molecular weight and becomes toxic to neurons. Recent studies have shown that compared with normal controls, patients with diabetes had higher CSF tau and α-synuclein levels (Pagano et al., 2018).

Both hyperphosphorylated tau (Stoothoff et al., 2009) and α-synuclein (Ordonez et al., 2018) can cause mitochondrial dysfunction and eventually lead to neuronal death. Mitochondria are essential for generating ATP through respiration and oxidative phosphorylation, and have a central role in apoptosis. Mitochondrial dysfunction is a key factor of cognitive deficits in neurodevelopmental disorders and neurodegenerative diseases (Sheng and Cai, 2012; Khacho et al., 2019). Growing evidence shows that even before the occurrence of AD symptoms or any detectable cognitive decline, mitochondrial dysfunction already happened (Kulic et al., 2011). Interaction between dysfunctional mitochondria and impaired insulin signaling can finally promote the development of AD in diabetic patients. This suggests that overlapping pathological mechanisms exists in neurodegenerative diseases and diabetes (Craft and Watson, 2004). Hence, antidiabetic agents might have potential benefits in the treatment of diabetic cognitive impairment.

**FIGURE 1:**
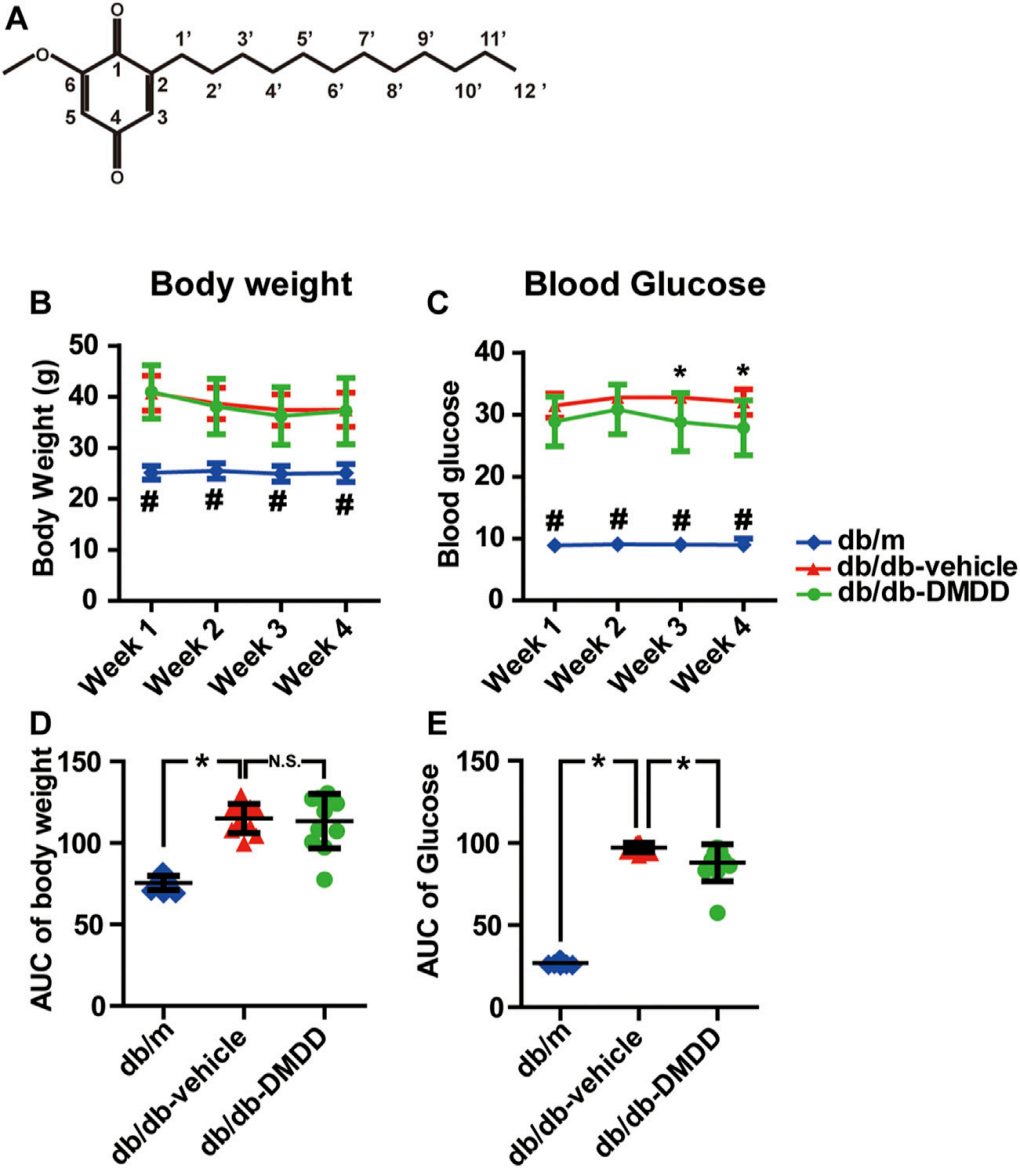
2-Dodecyl-6-methoxycyclohexa-2,5-diene-1,4-dione (DMDD) improved hyperglycemia in db/db mice. **(A)** Chemical structural diagrams of DMDD. **(B)** Body weights and **(D)** area under the curve (AUC) during 4 weeks of drug treatment. **(C)** Blood glucose and **(E)** AUC during drug treatment. All data are shown as means ± SD. *p < 0.05 for one-way ANOVA test.

Natural products are the main source of new drug compounds. Studies have shown that natural products and derived compounds can attenuate cognitive impairment and diabetes. 2-Dodecyl-6-methoxycyclohexa-2,5-diene-1,4-dione (DMDD) (Figure 1A) is an active compound, which was purified from the roots of *Averrhoa carambola* L. Previous studies in our lab have suggested that DMDD had an antidiabetic effect by decreasing AGE expression and downregulating the NFκB–TGFβ1 pathway in type 2 diabetic KKAy mice (Zheng et al., 2013). In addition, DMDD also protected against apoptosis in PC-12 pheochromocytoma cells *in vitro* and APP/PS1 Alzheimer’s disease mice *in vivo* (Wei et al., 2018). Although the therapeutic effect of DMDD on diabetes mellitus and Alzheimer’s disease were studied, the therapeutic potential of DMDD on diabetic cognitive impairment and its underlying mechanisms remains unclear. Here, we investigated the neuroprotective effect and possible mechanisms of DMDD on high-glucose-induced HT22 mouse hippocampal neuron injury and on diabetic db/db mice with impaired learning and memory.

## Materials and Methods

### Plant Material and Isolation of 2-Dodecyl-6-Methoxycyclohexa-2,5-Diene-1,4-Dione

2-Dodecyl-6-methoxycyclohexa-2,5-diene-1,4-dione (DMDD), a cyclohexanedione, was isolated and purified from *Averrhoa carambola* L., which is cultivated in tropical and subtropical regions. The roots of *Averrhoa carambola* L. were collected from Lingshan, Guangxi, China, and identified by Prof Maoxiang Lai (Traditional Chinese Medicine Research Institute of Guangxi). For *in vitro* assay, DMDD was dissolved in 100% DMSO as 20 mM stock solution. DMDD working solution was prepared by diluting appropriate stock solution in cell culture medium. The chemical structure of DMDD is shown in Figure 1A.

### Mice and Drug Treatments

Male 9-week-old db/db mice and age-matched heterozygous db/m control mice were bought from the Model Animal Research Center of Nanjing University (Nanjing, China). The mice were housed in a controlled environment (25 ± 2°C and 50 ± 5% relative humidity) with a 12-h/12-h day/night cycle. The mice were given free access to food and water. All animal experimental protocols were approved by the Animal Ethics Committee of Guangxi Medical University.

Db/db mice were randomly assigned to vehicle-treated db/db and DMDD-treated db/db group (10 mice per group) after 1 week of adaption. The wild-type db/m control mice were used as the control group (*n* = 10). Db/m control group and vehicle-treated db/db group were administrated intragastrically with 1% Tween 80 per day. The DMDD-treated db/db group was administrated intragastrically with 50 mg/ kg of DMDD per day. Drug treatment was performed once daily during the daytime for 28 successive days. Body weight and blood glucose were detected every 2 days. The animal behavior test was carried out before and after the drug treatment. At the end of the study, the mice were euthanized. Then the hippocampus were isolated and kept in liquid nitrogen for Western blotting, real-time PCR, and lncRNA microarray analysis.

### Y-Maze Test

**FIGURE 2:**
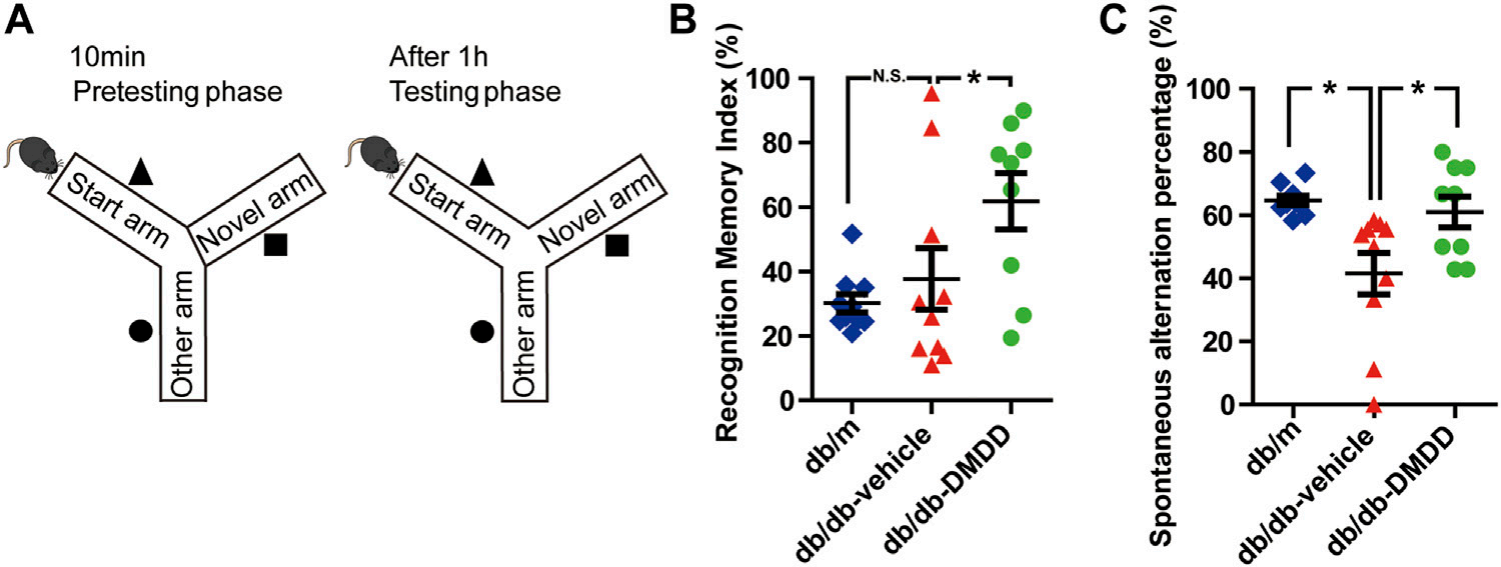
DMDD attenuated spatial working memory impairment in db/db mice. **(A)** Schematic diagram of the Y-maze. Figures representing recognition memory index **(B)** and spontaneous alternation percentage **(C)** in db/m-control (n = 10), vehicle-treated db/db (n = 10), and DMDD-treated db/db (n = 9) mice. Representing recognition memory index and spontaneous alternation percentage are shown by scatter plot. All data are shown as means ± SD. *p < 0.05 for one-way ANOVA test.

Y-maze test, a two-trial spatial memory task was designed to assess spatial memory in mice (Kraeuter et al., 2019). During the first trial (acquisition), the mouse was able to visit two arms (named as start arm and other arm) of the Y-maze for 10 min, and the third arm (named as new arm) was blocked by a door with the same color as the aisle. After the end of the first trial, the mouse was placed in a single cage. After 1 h, the second trial (retrieval) started. The door of the new arm was opened, and the mouse accessed freely all three arms for 5 min (Figure 2A). The number and the duration of visiting each arm were recorded for each trial. During the second trial, every entry different from the last two entries was regarded as a successful alternation. The following formula was used to calculate working memory: spontaneous alternation percentage (%) = [(number of successful alternations)/(total number of arm entries −2)] × 100%. Recognition memory index (%) = (time in the novel arm/time in all arms during the second trial) × 100%.

### Two-Object Novel Object Recognition

**FIGURE 3:**
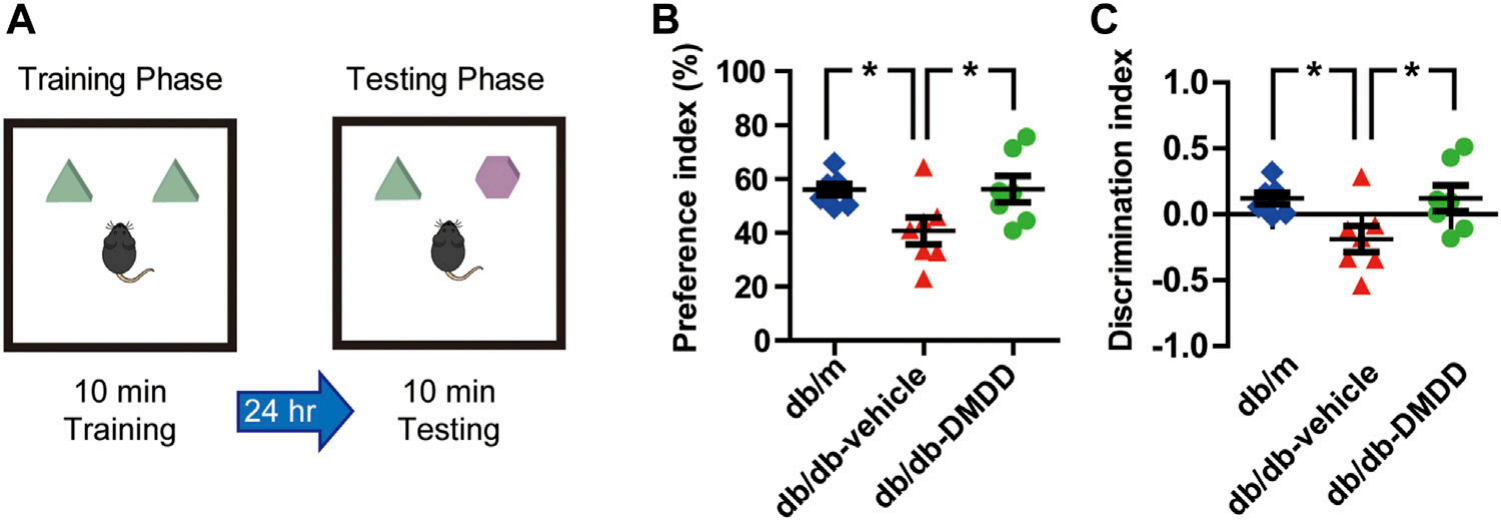
DMDD attenuated object recognition memory impairment in db/db mice. **(A)** Schematic diagram of the object recognition task during the training phase and testing phase. Preference index **(B)** and discrimination index **(C)** increased significantly in DMDD-treated db/db group compared with vehicle-treated db/db group during the testing phase. All data are shown as means ± SD. *p < 0.05 for one-way ANOVA test.

Novel object recognition task was executed using the following steps(Lueptow, 2017): 1) Habituation: the mouse was allowed to explore freely in the empty test tank for 10 min per day for 2 days. 2) Training: 24 h after habituation, two indistinguishable objects were placed in the symmetric quadrants of the arena, and then the mouse was put in the tank and allowed free exploration for 10 min. 3) Testing: 24 h after training, one of the previous objects was replaced with a new object at the same location. The mouse was again placed in the tank and allowed free exploration for 10 min. As illustrated in Figure 3A, the whole testing phase was recorded and analyzed by a behavioral analyzing system (Shanghai Xinruan Information Technology Co. Ltd). The following formula was used to calculate short-term memory: discrimination index = [(exploration time at novel object - exploration time at old object)/(exploration time at novel object + exploration time at old object)]. Preference index = exploration time at novel object/(exploration time at novel object + exploration time at old object).

### Cell Culture and Treatments

The HT22 mouse hippocampal neuron cells were obtained from GuangZhou Jennio Biotech Co., Ltd. The HT22 cell line was genotyped for identity and was tested routinely for *Mycoplasma* contamination. The HT22 cell was maintained in DMEM medium (Gibco) commonly with 10% FBS (Gibco) at 37°C in cell incubator with 5% CO_2_. Normal glucose (NG, 25 mM) DMEM medium contained 25 mM glucose. Additional 25 mM glucose was added into NG DMEM to prepare the high-glucose (HG, 50 mM) DMEM medium. Cells were treated with vehicle (DMSO) or DMDD for 48 h after incubation with NG or HG DMEM medium for 24 h.

### Cell Death and Viability Detection

The HT22 cells were seeded at 1.5 × 10^3^/well in a 96-well cell culture plate and then treated with HG medium as described above. Cell viability was measured by CellTiter 96^®^ AQueous one solution cell proliferation assay system (Promega) according to the instructions of the manufacturer. Cell viability or death rates were calculated by normalizing to the 0 μM DMDD or NG medium group. Apoptosis was detected by Hoechst 33342 (7 mM) and propidium iodide (2 mM) (Invitrogen). Images were captured by Olympus microscope.

### RNA Microarray Analysis

Shbio mouse lncRNA array V6.0 (4 × 180K, Shanghai Biotechnology Corporation) is designed for global profiling of mouse lncRNAs and mRNA transcripts and includes 64,221 lncRNA and 26,531 mRNA probes. Total RNA from mice hippocampus tissues and cell lines was extracted using RNeasy mini kit (Qiagen). RNA integrity was checked for an RIN number by an Agilent Bioanalyzer 2,100. For lncRNA microarray analysis, total RNA was amplified and labeled by Labeling Kit (Agilent technologies). Slides were hybridized with labeled cRNA and were scanned by Agilent Microarray Scanner. Microarray data were extracted by Feature Extraction software 12.0 (Agilent technologies) and normalized by Quantile algorithm. To reveal the potential biological processes associated with DMDD regulatory genes, Gene Ontology enrichment analysis and KEGG pathway analysis were performed based on significantly different expression genes and lncRNA target genes using clusterProfiler in R/bioconductor.

### Real-Time Quantitative PCR

Total RNA was reverse transcribed into cDNA using Reverse Transcription Kit (Thermo Fisher Scientific). qRT-PCR was performed using Power SYBR™ Green (Thermo Fisher Scientific) and ABI 7300 detection system (Applied Biosystems). qRT-PCR data were normalized to the expression of housekeeping gene GAPDH, and relative expressions were calculated using the 2^−ΔΔCt^ method. The oligonucleotide primers were shown as follows: Hif3α (sense: 5′-TCG​TGC​ATT​CTC​ATG​AGC​CC-3′, antisense: 5′-GTT​CCT​GGG​CTG​GAC​AGT​TT-3′), Mmp8 (sense: 5′-CCA​ATG​CCT​TCC​CAG​TAC​CTG​A-3′, antisesce: 5′-GGC​ATT​CCT​CGA​AGA​CCG​GAA​T-3′), Timp1 (sense: 5′- CTA​TAG​TGC​TGG​CTG​TGG​GG-3′, antisense: 5′-GGA​CCT​GAT​CCG​TCC​ACA​AA-3′), Fam187b (sense: 5′ ACC​AAC​CAG​CCG​TAC​TTT​GT3′, antisense: 5′-TTG​TCA​GCT​TCC​CAG​TGG​AC-3′). All tests were performed in triplicate, and the data were presented as mean ± SD.

### Microarray Data Collection

Gene expression datasets GDS810 (Blalock et al., 2004) and GDS2519 (Scherzer et al., 2008) were downloaded from the GEO database (https://www.ncbi.nlm.nih.gov/geo/) and analyzed by GEO2R (https://www.ncbi.nlm.nih.gov/geo/geo2r/). These datasets include AD (GDS810, brain hippocampi samples from nine healthy normal control and 22 ADs at various stages including 7 incipient,8 moderate, and 7 severe AD) and PD (GDS2519, blood samples from 22 healthy control and 50 PDs) with healthy control. These two microarray datasets were detected by the same platform (HG-U133A) Affymetrix Human Genome U133A Array.

### Molecular Docking

The 3D format of DMDD was obtained from the ZINC15 drug database (http://zinc15.docking.org/) (Sterling and Irwin, 2015). The x-ray crystal structure of Hif3α was obtained from the RCSB PDB protein databank (http://www.rcsb.org/). The water molecules and ligand molecules were removed from the crystal structures by Discovery Studio 4.5 Client before the docking simulation began. Docking simulations of DMDD and Hif3α was carried out by PyRx software. Interaction energies were calculated for predicting docking positions and selecting the binding pose that had the lowest binding energy (kcal mol^−1^). Results were visualized and analyzed using Discovery Studio 4.5 Client.

### Western Blot Analysis

Cells or hippocampus tissues were homogenized in RIPA buffer (SIGMA). Concentration of protein was measured using BCA Assay Kit (Beyotime). Equivalent protein was separated by polyacrylamide gel electrophoresis and transferred to polyvinylidene difluoride membranes (Millipore). After blocking the membranes with 5% nonfat milk, the membranes were incubated overnight at 4°C with primary antibodies against Tau phospho Serine 199/202 (1:1,000, Cat. AB9674, Millipore), Tau-1 (1:1,000, Cat. MAB3420, Millipore), phospho-α-Synuclein (Ser129) (1:1,000, Cat. MABN826, Millipore), α-Synuclein (1:1,000, Cat. 2,647, Cell signaling), HIF-3 alpha (1:1,000, Cat. NB100-2,529, Novus), and gapdh (1:1,000, Cat. 2,118, Cell signaling) overnight at 4°C. Then membranes were incubated with HRP-conjugated secondary antibody (1:2,000 dilution) and detected using Chemiluminescent Substrate (Thermo Scientific). Band intensity was quantified by ImageJ.

### Statistical Analysis

Data were analyzed using GraphPad Prism 5 and SPSS 19.0 software. To identify gene expression patterns, an unsupervised hierarchical cluster analysis using un-centered average linkage was performed using Cluster v3.0 after median-centering genes and arrays. Heat maps were visualized using Tree View software. The data were analyzed by one-way ANOVA followed by Tuckey’s tests. A value of *p* < 0.05 was considered significant.

## Results

### 2-Dodecyl-6-Methoxycyclohexa-2,5-Diene-1,4-Dione Treatment Improved Hyperglycemia in db/db Mice

Compared with db/m control mice, the blood glucose levels (Figure 1C) and the total area under the curve (AUC) of the blood glucose (Figure 1E) were significantly increased in vehicle-treated db/db mice (*p* < 0.05). DMDD treatment (50 mg/ kg) significantly reduced hyperglycemia (*p* < 0.05) in DMDD-treated db/db mice at weeks 3 and 4 (Figure 1C). The AUC of blood glucose was significantly decreased after DMDD treatment (*p* < 0.05) (Figure 1E). A marked increase in vehicle-treated db/db mice body weight was observed at the first week compared with db/m control mice (Figures 1B,D). However, DMDD treatment had no meaningful effect on body weight in DMDD-treated db/db mice (Figures 1B,D).

### 2-Dodecyl-6-Methoxycyclohexa-2,5-Diene-1,4-Dione Treatment Attenuated Spatial Working Memory Impairment in db/db Mice

The spatial working memory of mice was investigated using Y maze (Figure 2A). As shown in Figure 2C, compared with the db/m-control mice, the spontaneous alternation percentage was significantly decreased (*p* < 0.05), and DMDD treatment reversed this change (*p* < 0.05) in vehicle-treated db/db mice. Meanwhile, the percentage of duration in the novel arm (measured as recognition memory index) was significantly higher in DMDD-treated db/db mice than in the vehicle-treated db/db mice (*p* < 0.05) (Figure 2B). These results suggested that DMDD treatment had significant positive effects on the spontaneous alternation and improved spatial working memory in db/db mice.

### 2-Dodecyl-6-Methoxycyclohexa-2,5-Diene-1,4-Dione Treatment Attenuated Object Recognition Memory Impairment in db/db Mice

We performed novel object recognition (NOR) test to examine the object recognition memory and short-term memory of mice (Figure 3A). During the training phase, no significant difference was found among each group. During the testing phase, preference and discrimination index of vehicle-treated db/db mice was decreased significantly compared with that of db/m-control mice (*p* < 0.05) (Figures 3B,C), which suggested that vehicle-treated db/db mice had decreased abilities to recognize a novel object. DMDD treatment significantly improved the preference and discrimination index (*p* < 0.05) (Figures 3B,C). Collectively, our results showed that administration of DMDD attenuated object recognition memory impairment in db/db mice.

### 2-Dodecyl-6-Methoxycyclohexa-2,5-Diene-1,4-Dione Treatment Reduced Tau-1 in the Hippocampi of db/db Mice

**FIGURE 4:**
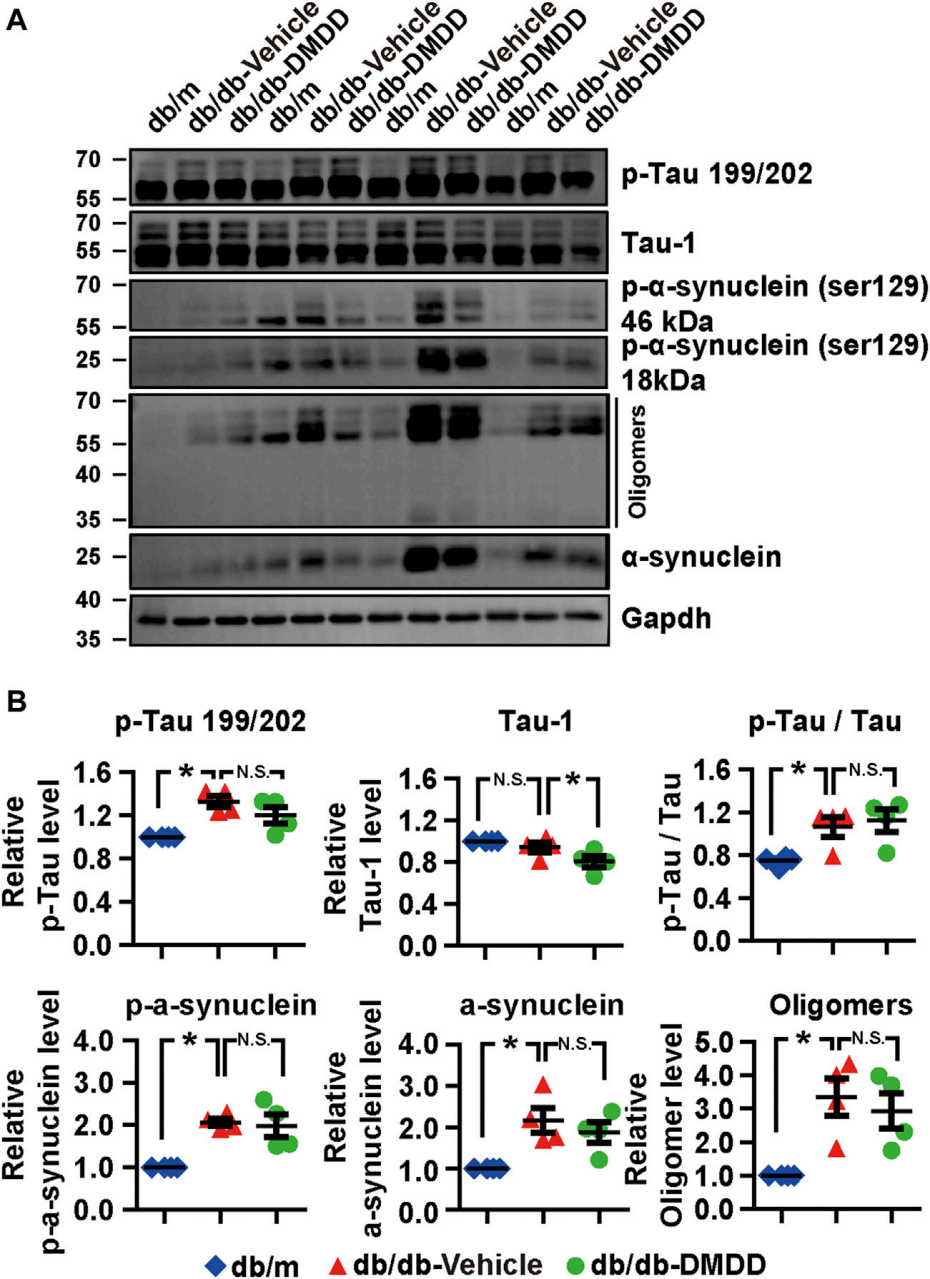
Neurodegeneration disease-related protein expression was reduced after DMDD treatment in the type 2 diabetes (T2D) db/db mice hippocampus. **(A)** The expressions of neurodegeneration disease-related proteins in the hippocampi of db/m-control mice (*n* = 4), vehicle-treated db/db (*n* = 4), and DMDD-treated db/db (*n* = 4) mice were analyzed by Western immunoblotting. **(B)** The relative expression of proteins was standardized by gapdh and db/m-control group for each sample. Data are expressed as means ± SD of four independent samples, *p < 0.05 for one-way ANOVA test.

Hyperphosphorylation of tau and α-synuclein is an important feature of neurodegenerative disease. We further explored whether DMDD treatment could influence the expression of tau and α-synuclein in the hippocampi of db/db mice. As shown in Figures 4A,B, compared with db/m control mice, tau proteins phosphorylated at Serine 199/202, the p-Tau/Tau ratio, p-α-synuclein, α-synuclein, and oligomers of α-synuclein were markedly increased in the hippocampus of vehicle-treated db/db mice (*p* < 0.05). The expression of p-tau 199/202, the ratio of p-Tau 199/202/total tau, p-α-synuclein (Ser129), α-synuclein, and oligomers of α-synuclein were increased by 1.35-, 1.50-, 2.05-, 2.10-, and 3.34-fold in db/db mice compared with db/m control mice (*p* < 0.05). After DMDD treatment, the expression of Tau-1 was significantly decreased (*p* < 0.05) in db/db mice. Although p-α-synuclein (Ser129, 18, and 46 kDa), α-synuclein, and its oligomers protein levels showed a decreasing trend after DMDD treatment, there was no significant difference between db/db-vehicle and db/db-DMDD group.

### lncRNAs and mRNAs Expressed Profiles in the 2-Dodecyl-6-Methoxycyclohexa-2,5-Diene-1,4-Dione-Treated db/db Mice

**FIGURE 5:**
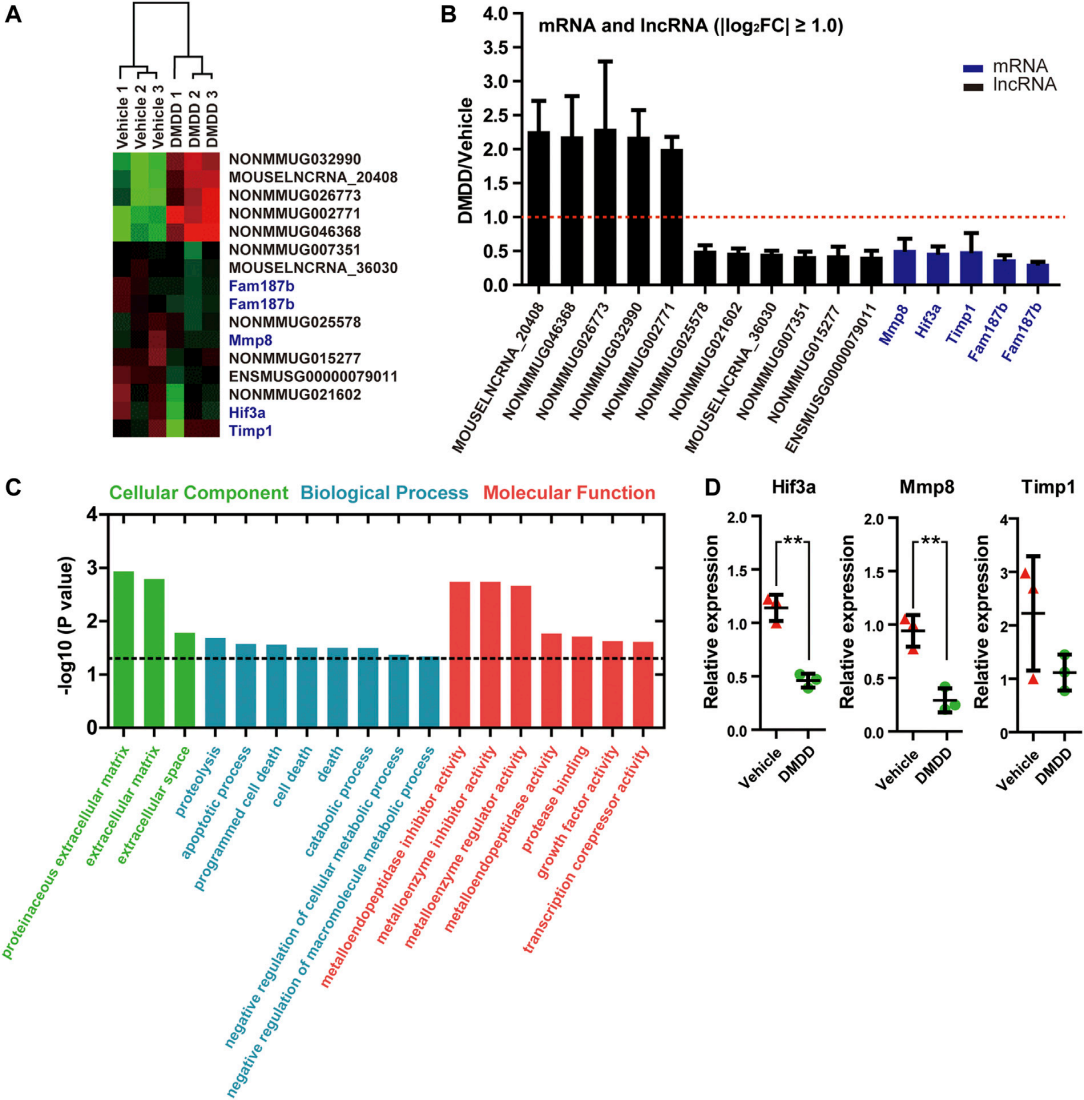
mRNAs and lncRNAs regulations in the hippocampi of DMDD-treated db/db mice. **(A)** Heat maps showing 16 different expressed genes between vehicle (*n* = 3) and DMDD (*n* = 3)-treated db/db mice, including 11 lncRNAs and5 mRNAs (fold change >2.0 and p < 0.05). Columns represent samples, rows represent lncRNAs (black) and mRNAs (blue), red means high expression, and green means low expression. **(B)** Bar graph showing altered mRNAs and lncRNAs in DMDD-treated T2D db/db mice hippocampus (*n* = 3) using microarray analysis. Vehicle group (*n* = 3) is represented by red dashed line. **(C)** Gene Ontology enrichment analysis of the different expressions mRNAs in vehicle (*n* = 3) and DMDD (*n* = 3)-treated db/db mice. A value of *p* equal to 0.05 is represented by a black dashed line. **(D)** Expressions of hypoxia-inducible factor-3α (Hif3α), Mmp8, and Timp1 were verified in three pairs of vehicle and DMDD-treated db/db mice by qRT-PCR. All data are shown as means ± SD. * *p* < 0.05, ** *p* < 0.01 for Student’s t-test.

In order to reveal the underlying mechanisms and potential targets of DMDD treatment in db/db mice, we performed RNA array on db/db-vehicle (*n* = 3) and db/db-DMDD (*n* = 3) mice hippocampal samples to find the genes that changed after DMDD treatment. Results showed that a total of 34 mRNAs and 120 lncRNAs probes had significantly different expression in hippocampi of DMDD treated mice compared with that of db/db-vehicle mice (*p* < 0.05, |log2 (fold change)| ≥ 0.5). Of these, 12 mRNAs and 59 lncRNAs were upregulated, and 22 mRNAs and 61 lncRNAs were downregulated in DMDD-treated db/db mice (Supplementary Table S1). Further analysis identified 11 lncRNAs and 4 mRNAs as candidate genes (*p* < 0.05, |log2 (fold change)| ≥ 1.00) (Figures 5A,B). We predicted cis and trans mRNA targets of these 11 lncRNAs by the sequence of lncRNA (Supplementary Table S2). Unfortunately, there was no intersection between the candidate mRNAs and the predicted lncRNA targets. To understand the function of candidate mRNAs (Mmp8, Hif3α, Timp1, and Fam187b), Gene Ontology enrichment analysis was performed and three cellular components were identified, including proteinaceous extracellular matrix (*p* = 0.0012), extracellular matrix (*p* = 0.0016), and extracellular space (*p* = 0.017). Eight biological processes were identified including proteolysis (*p* = 0.021), apoptotic process (*p* = 0.027), programmed cell death (*p* = 0.028), cell death (*p* = 0.031), death (*p* = 0.031), catabolic process (*p* = 0.032), negative regulation of cellular metabolic process (*p* = 0.043) and negative regulation of macromolecule metabolic process (*p* = 0.046). Molecular functions were identified including metalloendopeptidase inhibitor activity (*p* = 0.0018), metalloenzyme inhibitor activity (*p* = 0.0018), metalloenzyme regulator activity (*p* = 0.021), metalloendopeptidase activity (*p* = 0.017), protease binding (*p* = 0.020), growth factor activity (*p* = 0.024), and transcription corepressor activity (*p* = 0.024) (Figure 5C). We validated Mmp8, Hif3α, Timp1, and Fam187b expression in hippocampi of vehicle and DMDD-treated db/db mice by independent real-time PCR. The result showed that Hif3α (*p* = 0.0033) and Mmp8 (*p* = 0.0047) were significantly lower in db/db-DMDD mice than in db/db-vehicle mice. Although Timp1 decreased after DMDD treatment, there was no significant difference (*p* > 0.05) (Figure 5D). The Ct value of Fam187b was too low to detect (Ct value >40). Based on these results, we chose Mmp8 and Hif3α as DMDD potential targets for the following study.

### Hypoxia-Inducible Factor-3α mRNA Expression was Significantly Increased in Alzheimer’s Disease and Parkinson’s Disease

**FIGURE 6:**
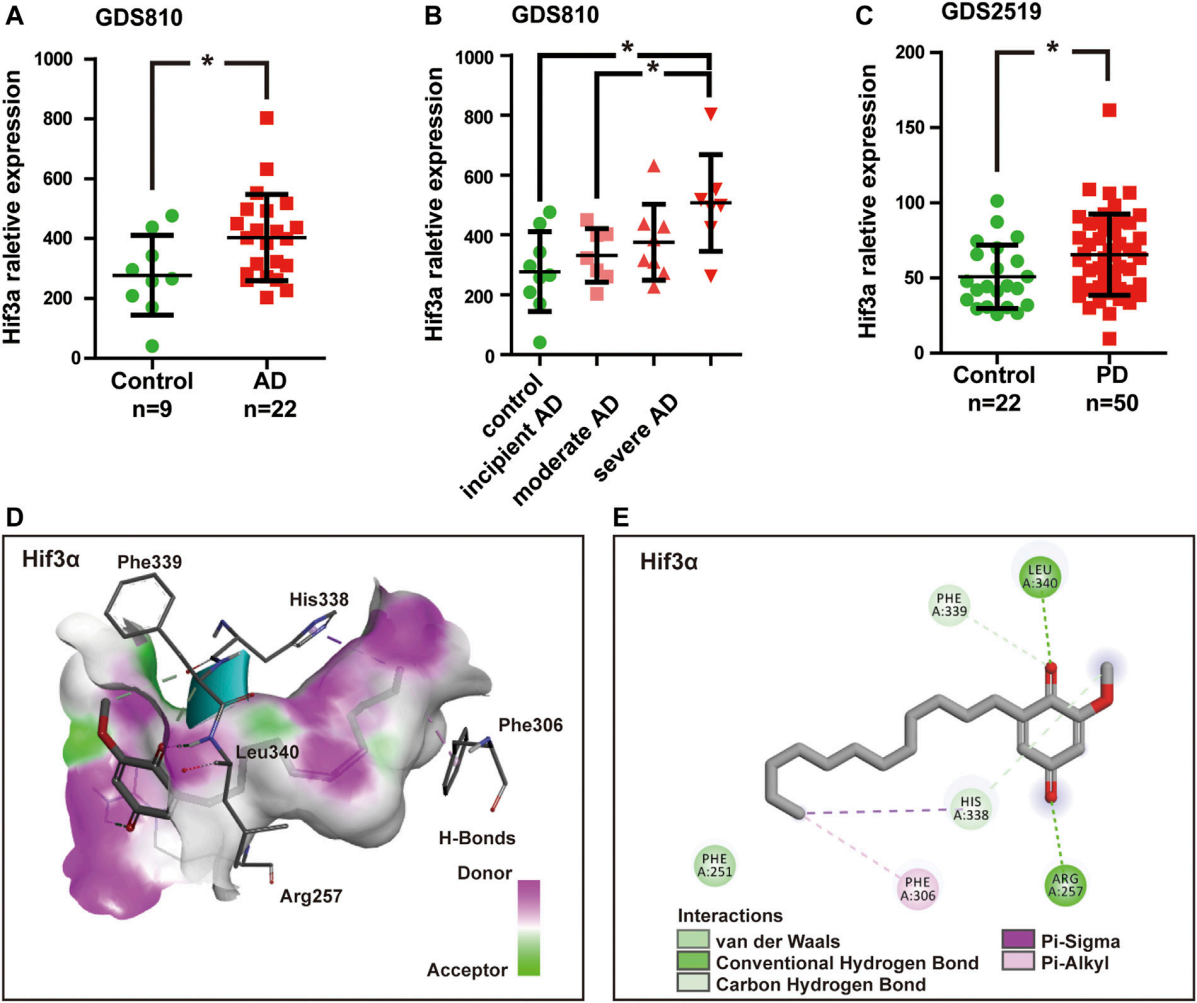
Hif3a mRNA expression was significantly increased in Alzheimer’s disease (AD) and Parkinson’s disease (PD). **(A)** Hif3a expression levels in AD and healthy control hippocampi in GDS810 data set (9 healthy control vs. 22 ADs). **(B)** Hif3a expression level was significantly higher in severe AD than in incipient AD in GDS810 data set (9 healthy control vs. 22 ADs at various stages including 7 incipient, 8 moderate, and 7 severe ADs). **(C)** Hif3a expression levels in PD and healthy control blood samples in GDS2519 data set (22 healthy control *vs*. 50 PDs). **(D)** DMDD binding with the pocket of Hif3a is composed of hydrogen bonds and the interaction pattern of DMDD with the residues. **(E)** A 2D diagram between the Hif3a and residues.*p < 0.05 for Student’s t-test.

Due to the fact that T2D and neurodegeneration disease shared the overlapping pathophysiological mechanisms and pathways, we detected Mmp8 and Hif3α mRNA expression value in AD and PD samples by analyzing gene expression datasets in GEO. Two related datasets were downloaded and analyzed, including expression profiling of AD brain hippocampi (GDS810, 9 healthy normal control and 22 ADs at various stages including 7 incipient, 8 moderate, and 7 severe AD) (Blalock et al., 2004) and expression profiling of PD blood (GDS2519, 22 healthy control, and 50 PDs) (Scherzer et al., 2008). The results showed that no significant difference was found for Mmp8 expression between healthy control and AD or PD (*p* > 0.05). However, Hif3α was significantly increased in AD and PD compared with healthy control (*p* < 0.05, Figures 6A,C). Remarkably, the expression of Hif3α was significantly higher in severe AD than in incipient AD (*p* < 0.05, Figure 6B).

A molecular docking model was constructed to further explore the mechanism of interaction between DMDD and Hif3α (PDB, 4WN5). Processing of the DMDD included energy minimized. The refinement of structure of DMDD was used for the dock. PyRx was used for the docking studies. The docked conformation corresponding to the lowest binding energy was selected as the most probable binding conformation. The result showed that DMDD could stay in a binding pocket surrounded by Hif3α amino acid Arg257, Phe306, His338, Phe339, and Leu340 (Figures 6D,E). These suggested that Hif3a was not only a potential target of DMDD, but also a diagnostic biomarker of AD and PD.

### 2-Dodecyl-6-Methoxycyclohexa-2,5-Diene-1,4-Dione Protects Mouse Hippocampal HT22 Cells From Hyperglycemia-Induced Injury

**FIGURE 7:**
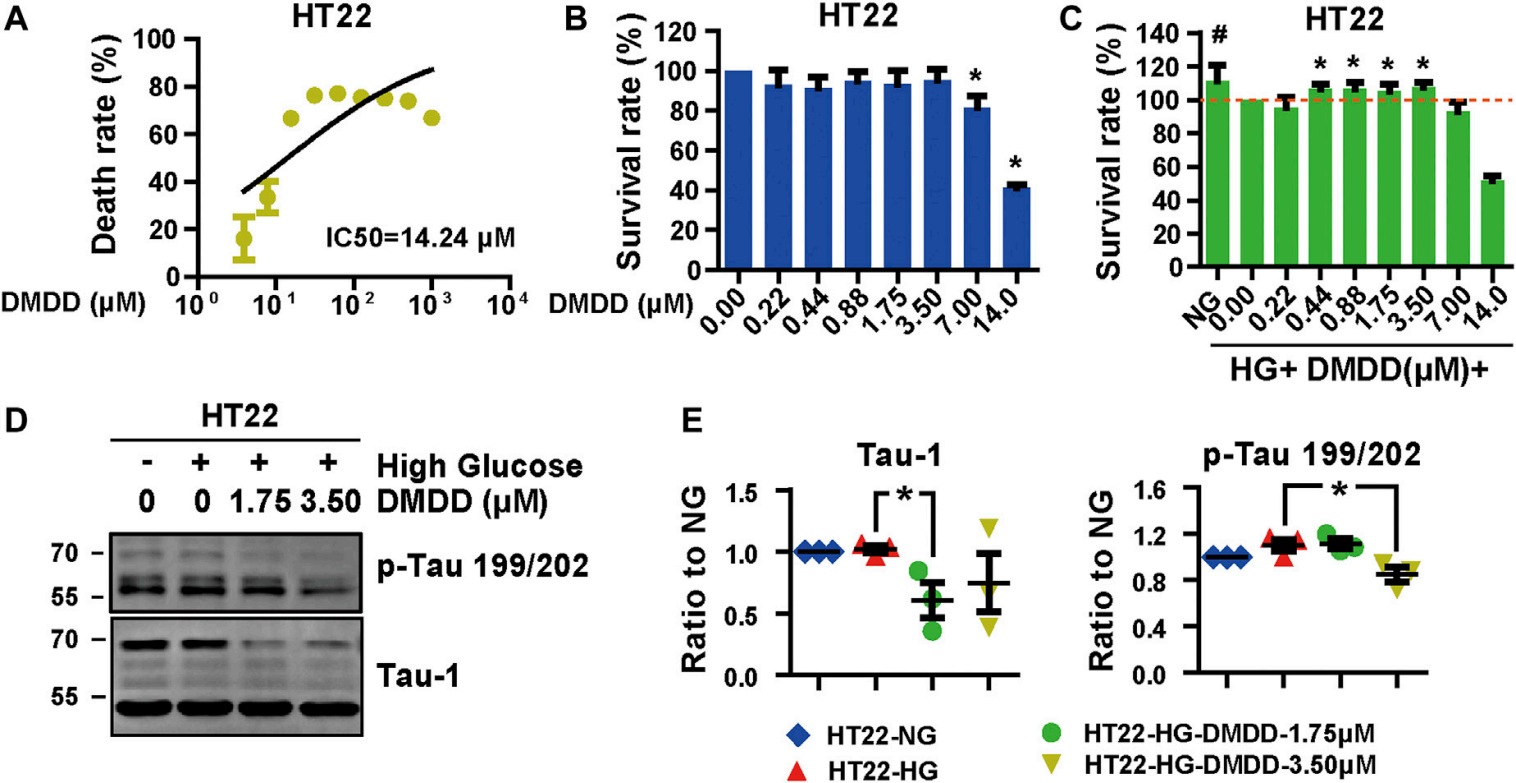
DMDD reduced neurodegeneration disease-related protein expression in HT22 cells. **(A)** The dose-effective curve of DMDD on HT22 cells. **(B**) DMDD treatment had no obvious cytotoxicity with concentrations under 3.50 μM in HT22 cell. **(C)** The protection effect of DMDD on the high glucose-treated HT22 cells. **(D)** AD-related proteins were tested by Western blotting. **(E)** Relative expression of proteins was standardized by gapdh and HT22-NG group for each sample. Data are expressed as means ± SD, *p < 0.05 for Student’s t-test.

In order to further confirm the neuroprotection of DMDD, the cell injury model was established by high-glucose treatment in HT22 cells. The protective effects of DMDD were investigated by MTS assay. The result showed that the apparent cytotoxicity could be detected when HT22 cells were cultured for 48 h with high concentrations of DMDD (7.00 μM) (Figures 7A,B). However, there was no obvious difference in cell viability between the DMDD-treated groups of different low concentrations (0.22, 0.44, 0.88, 1.75, and 3.50 μM) and the vehicle group (*p* > 0.05) (Figure 7B). As shown in Figure 7C, the cell viability of HT22 cells was significantly decreased after high-glucose treatment for 48 h (*p* < 0.05), and the administration of DMDD at concentrations of 0.44, 0.88, 1.75, and 3.50 μM obviously inhibited the decrease in cell viability induced by high glucose in HT22 cells (*p* < 0.05) (Figure 7C). In addition, the treatment with 1.75 μM of DMDD reduced Tau-1 protein levels (*p* < 0.05) and treatment with 3.50 μM of DMDD reduced p-Tau 199/202 protein levels (*p* < 0.05) compared with the high glucose-treated HT22 cells (Figures 7D,E). These results indicated that DMDD treatment had a protective effect on high glucose-induced neural damage.

### 2-Dodecyl-6-Methoxycyclohexa-2,5-Diene-1,4-Dione Protected Against High Glucose-Induced Apoptosis in HT22 Cells and the Hippocampi of Db/Db Mice

**FIGURE 8:**
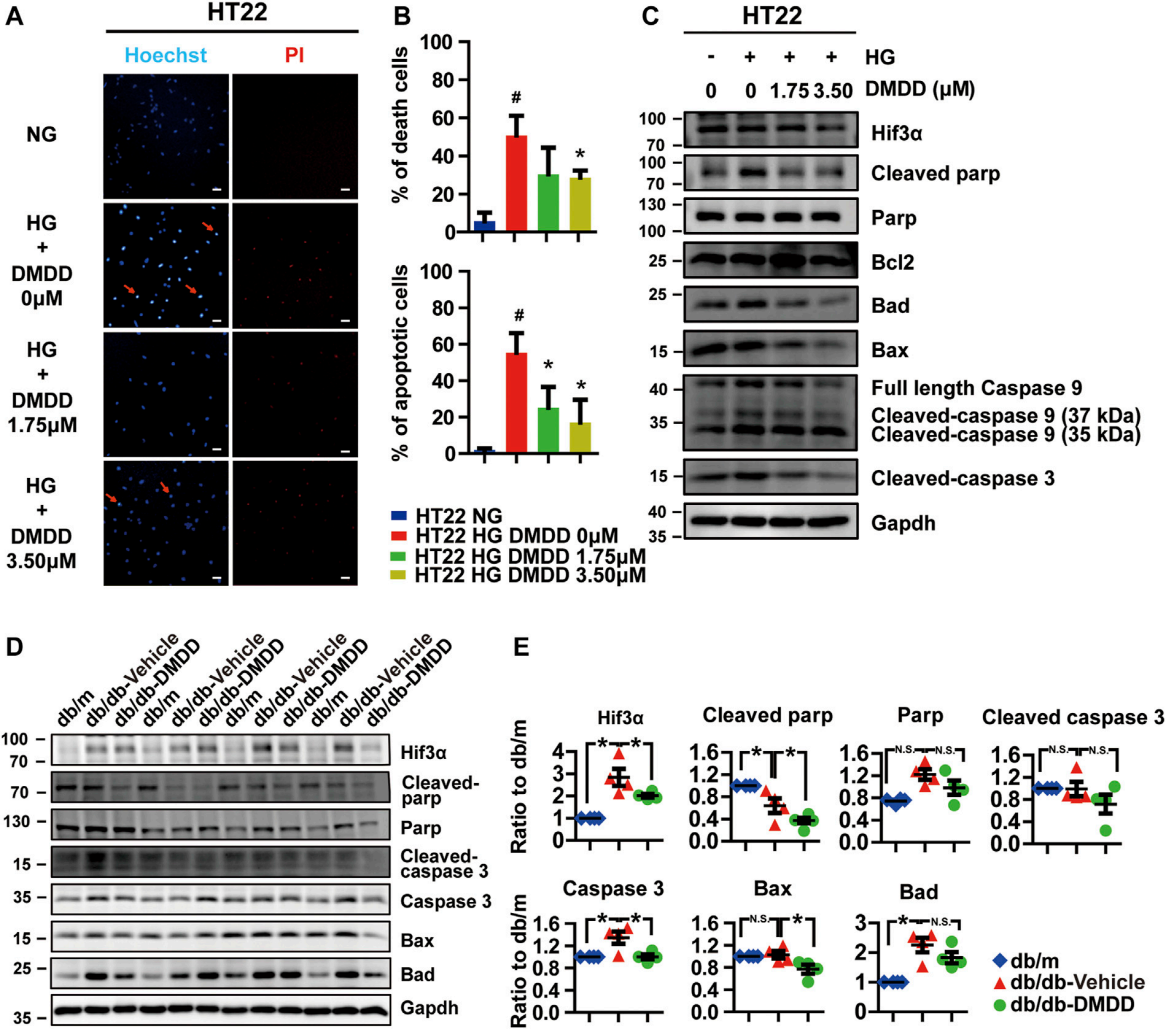
DMDD prevented high glucose-induced apoptosis in HT22 cells and hippocampi of T2D db/db mice. **(A)** Images of Hoechst 33342 and PI staining in HT22 cells pre-incubated with DMDD (0, 1.75, 3.50 μM) for 48 h in normal glucose (NG) or high-glucose (HG) medium. Scale bars, 20 mm. **(B)** Quantification of dead and apoptotic cells (*n* = 4). **(C)** HT22 cells were incubated with DMDD (0, 1.75, 3.50 μM) for 48 h in NG or HG medium. Hif3α and apoptosis-related proteins were detected by Western blot. **(D)** The expressions of HIF3α and apoptosis-related protein in db/m-control mice (*n* = 4), vehicle-treated db/db (*n* = 4), and DMDD-treated db/db (*n* = 4) mice hippocampus tissues were measured by Western blotting. **(E)** Relative expression of proteins was standardized by gapdh and db/m-control group for each sample. Data are expressed as means ± SD, * *p* < 0.05 for one-way ANOVA test.

Considering that Hif3a is potential target for DMDD and is also a pro-apoptotic factor (Torii et al., 2011), we performed Hoechst and PI staining to evaluate the potential effect of DMDD on high glucose-induced apoptosis in HT22 cells. The results showed that cell apoptosis and death were significantly increased after high glucose treatment in HT22 cells, whereas DMDD prevented high glucose induced-apoptosis and death in HT22 cells (Figures 8A,B). The expression levels of apoptosis markers further validated the above results. Western blot analysis showed that high glucose upregulated the expression of Hif3a, cleaved parp, Bad, Bax, Caspase 9, cleaved Caspase 9 (37 kDa), and cleaved Caspase3 in HT22 cells (Figure 8C). DMDD treatment inhibited high glucose-induced expression of Hif3a, cleaved parp, Bad, Bax, caspase 9, cleaved caspase 9 (37 kDa), and cleaved caspase 3 in HT22 cells (Figure 8C). Moreover, DMDD exerts a dose-dependent effect on the expression of Hif3a, Bad, Bax, caspase 9, cleaved caspase 9 (37 kDa), and cleaved caspase 3 (Figure 8C). Similar results were also demonstrated in the hippocampi of db/db mice. As shown in Figures 8D,E, there were significantly higher Hif3a, caspase 3, and Bad expression in vehicle-treated db/db group than in db/m group (*p* < 0.05). However, the expression levels of Hif3a, cleaved Parp, caspase 3, and Bax were significantly reduced in DMDD-treated db/db group compared with db/db-vehicle group (*p* < 0.05). These results indicated that DMDD treatment decreased pro-apoptotic factor Hif3a expression and protected neurons from high glucose-induced apoptosis *in vivo* and *in vitro*.

## Discussion

Type 2 diabetes mellitus (T2DM) is a progressive, chronic disease characterized by hyperglycemia and affects millions of people worldwide. The high incidence of neurodegenerative disease in patients with T2DM is supported by epidemiological data, and it is well recognized that T2DM is an independent risk factor of cognitive impairment (Sims-Robinson et al., 2010). In fact, mounting evidence showed that insulin resistance and deficiency could lead to structural and functional alterations in the brain. This suggests that overlapping pathological mechanisms exist in neurodegenerative diseases and diabetes (Craft and Watson, 2004). Hence, antidiabetic agents might have potential therapeutic benefits for the treatment of diabetic cognitive impairment.

2-Dodecyl-6-methoxycyclohexa-2,5-diene-1,4-dione (DMDD), an active compound was purified from the roots of *Averrhoa carambola L.* Previous studies have reported that DMDD improves cognitive impairment by protected against neuronal apoptosis in APP/PS1 Alzheimer’s disease mice (Wei et al., 2018). In addition to anticognitive impairment activity, DMDD had an antidiabetic activity by decreasing AGE expression and downregulating key genes in the NFκB–TGFβ1 pathway in KKAy mice. DMDD alleviates diabetic nephropathy by mitigating kidney damage and inflammation *via* the inhibition of the TLR4/MyD88/NF-κB signaling pathway (Lu et al., 2019). Notably, the NF-κB signaling pathway plays a critical role across many cellular processes, especially neuronal development (Garcia-Garcia et al., 2021). Suppression of the NF-κβ signaling pathway can prevent diabetes-associated cognitive impairment (Kuhad et al., 2009). Although therapeutic actions of DMDD on diabetes mellitus and Alzheimer’s disease were studied, the therapeutic potential of DMDD on diabetic cognitive impairment and its underlying mechanisms remains unclear.

Emerging evidence showed that the pathogenesis of AD, PD, and T2DM are synergistic (Moran et al., 2015; Pagano et al., 2018). Abnormal insulin level in T2DM promotes Lewy body (composed of α-synuclein) formation, tau phosphorylation, and ultimately leads to diabetic cognitive impairment (Sato and Morishita, 2014). Therefore, it is particularly important to choose a suitable animal model for studying diabetic cognitive impairment. Several animal models are used to investigate the effect of spontaneous type 2 diabetes on neurodegenerative disease. Most of them are characterized by leptin impairment. Leptin plays a key role in immunity, regulation of insulin secretion, and hippocampal behavioral deficits (Bjørbaek and Kahn, 2004; Yadav et al., 2013; Platt et al., 2016). Studies showed that the db/db mouse model, which carries mutated leptin receptor, exhibited metabolic syndrome of T2DM (such as hyperglycemia, overweight, dyslipidemia, insulin resistance, and inflammation), also accompanied with cognition impairment symptoms (Yermakov et al., 2019). In the present study, we observed significant hyperglycemia, excessive body weight, spatial working memory impairment, and object recognition memory impairment in db/db mice compared with db/m control mice. DMDD treatment improves hyperglycemia and learning memory impairment in db/db mice. In terms of pathological mechanism, tau and α-synuclein, which are the pathologic characteristics of AD and PD, were significantly upregulated in the hippocampus of db/db mice. DMDD treatment reduced the abnormal protein expressions of Tau-1 and α-synuclein in the hippocampi of db/db mice. These results confirmed that the db/db mice model could be effectively applied to diabetic cognitive impairment study. Furthermore, DMDD protects db/db mice from hyperglycemia and neural damage by decreasing the expression of Tau-1 and α-synuclein.

To date, only a few mRNAs and lncRNAs have been reported to be dysregulated and functionally characterized in diabetic cognitive impairment. Microarray provides a comprehensive assessment of expressed probes and allows identification of mRNAs and lncRNAs with diagnostic, prognostic, and functional potential in diabetic cognitive impairment (Ci et al., 2018; Santiago et al., 2019). To find the potential target genes of DMDD and cell signaling associated with diabetic cognitive impairment treatment, we test the gene expression profile in the hippocampi of db/db mice by RNA microarray. Eleven lncRNAs and four mRNAs had significantly different expressions in the hippocampi of DMDD-treated mice compared with db/db-vehicle mice. In order to identify molecular mechanisms of lncRNA, we predicted mRNA targets of these 11 lncRNAs by the sequence of lncRNA. Unfortunately, there was no intersection between the candidate mRNAs and the predicted lncRNA targets. GO analysis indicated that the potential target mRNAs of DMDD (Mmp8, Hif3α, Timp1, and Fam187b) were correlated with proteolysis, apoptotic process, programmed cell death, protease binding, and other important biological processes. Among them, defective proteolysis (Homma and Fujii, 2020), apoptosis (Li et al., 2020), and cell death (Nguyen et al., 2020) are closely related to neurodegenerative and metabolic diseases. We validated the potential target mRNA expression of DMDD by independent real-time PCR. The results showed that Hif3α and Mmp8 were significantly lower in the hippocampi of DMDD-treated db/db mice than in that of db/db-vehicle mice. These mRNAs could potentially serve as DMDD targets in diabetic cognitive impairment treatment.

Hypoxia-inducible factor-3α (Hif3a) is member of hypoxia-inducible factors (HIFs). As transcriptional factors, the HIF family has a vital role in transcriptional response to hypoxic stress by regulating different target genes. Compared with HIF1A and HIF2A, much less is known about HIF3A. This is, in part, due to the existence of multiple HIF3A variants that made it challenging to elucidate HIF3A functions. Recently, studies have shown that HIF3A expression and methylation are related to adipose tissue dysfunction (Pfeiffer et al., 2016), insulin resistance (Main et al., 2016), and gestational diabetes mellitus (Zhang et al., 2019). In addition, researchers also noted that microglial cells expressed HIF3A under inflammatory conditions, and hypoxia treatment resulted in a marked upregulation of HIF3A mRNA levels in the adult rat cortex, hippocampus, lungs, kidneys, and myocardial tissue (Heidbreder et al., 2003). These pathophysiological events are believed to be key factors contributing to neurodegeneration disease and diabetes mellitus progression. Significantly, a splicing variant of HIF3A, named IPAS (Makino et al., 2002; Torii et al., 2011; Kasai et al., 2017), has recently been found to act as a pro-apoptotic factor in the mitochondria by binding directly to the pro-survival Bcl-2 family and activating caspase 3 in PC12 cells. In addition, a previous study indicated that IPAS might take part in the pathogenesis of both sporadic and familial PD by acting as a substrate of PINK1 and Parkin and mediating neuronal cell death (Torii et al., 2015). In this study, Hif3a was further validated for its expression in two independent AD and PD microarray data sets. Moreover, we found that Hif3α was significantly increased in the hippocampi of AD patients and the blood of PD patients compared with that of healthy control, and related to the severity of AD. Then we conducted molecular docking between DMDD and Hif3α. The result showed that DMDD could stay in a binding pocket surrounded by Hif3α amino acid Arg257, Phe306, His338, Phe339, and Leu340. The mRNA and protein levels of Hif3α were upregulated in the hippocampi of db/db mice and high glucose-treated HT22 cells. It suggested that Hif3a was not only a potential target of DMDD to protect neurons from damage, but also a diagnostic biomarker of neurodegenerative disease.

Apoptosis plays a crucial role in diabetes-induced cognitive and memory impairment. Mitochondrial dysfunction is one of the main causes of apoptosis and is also a key factor of cognitive deficits in neurodevelopmental disorders and neurodegenerative diseases (Cheng et al., 2019). Growing evidence showed that even before the occurrence of AD pathological symptoms or any detectable cognitive decline, there are mitochondrial dysfunctions that happened (Iglesias-Gato et al., 2016). Interaction between dysfunctional mitochondria and impaired insulin signaling can finally promote the development of AD in diabetic patients (Jadiya et al., 2019). When mitochondrial dysfunction happened, antiapoptotic proteins Bcl-2 and Bcl-xL participated in this process by controlling mitochondrial permeability. They exist in the outer mitochondrial membrane and inhibit the release of pro-apoptotic protein cytochrome c from mitochondria. Following death signal, the pro-apoptotic proteins Bad, Bid, Bax, and Bim, which exist in the cytosol, will be transferred to the mitochondria, promote the release of cytochrome c to the cytosol, activate the caspase cascade, and lead to cell death (Pradelli et al., 2010; Ola et al., 2011). Apoptosis caused by mitochondrial dysfunction has been implicated in the pathogenesis of diseases characterized by excessive cell death, such as neurodegenerative diseases and diabetes (Quintanilla et al., 2009). Hif3a, as a pro-apoptotic protein, can block the interaction between Bcl-xL and Bax, and inhibits the antiapoptosis function of Bcl-xL (Torii et al., 2011). In this study, we found that cell apoptosis and death were significantly increased after high glucose treatment, and DMDD prevents high glucose-induced apoptosis and death in HT22 cells. Hif3a and apoptosis-related proteins cleaved parp, Bad, Bax, caspase 9, cleaved caspase 9, and cleaved caspase 3 were upregulated after high-glucose treatment. DMDD treatment inhibited high glucose-induced expression of Hif3a, cleaved parp, Bad, Bax, caspase 9, cleaved caspase 9, and cleaved caspase 3 *in vivo* and *in vitro*. These results indicated that DMDD treatment protected neurons from high glucose-induced apoptosis by regulating apoptosis-related protein Hif3α. However, the function and mechanism of Hif3a in the pathogenesis of high glucose-induced cognitive deficits is still unknown, and further research is needed.

## Conclusion

We studied the neuroprotective effects of DMDD to high glucose-induced HT22 hippocampal neuron cell injury *in vitro* and learning memory impairment of db/db mice *in vivo*. The results indicated that DMDD treatment reduced the abnormal protein expressions of Tau-1 in the hippocampi of db/db mice. Moreover, DMDD treatment inhibited hyperglycemia-induced apoptosis through inhibiting the pro-apoptotic protein Hif3a, cleaved parp, caspase 3, and Bax in hippocampal neurons. Our findings suggest that DMDD has the potential to prevent and treat cognitive impairment in people with diabetes.

## Data Availability

The datasets presented in this study can be found in online repositories. The names of the repository/repositories and accession number(s) can be found below: https://www.ncbi.nlm.nih.gov/geo/query/acc.cgi?acc=GSE174378

## References

[B1] Ashraf G. M. Greig N. H. Khan T. A. Hassan I. Tabrez S. Shakil S. (2014). Protein Misfolding and Aggregation in Alzheimer's Disease and Type 2 Diabetes Mellitus. CNS Neurol. Disord. Drug Targets 13, 1280–1293. 10.2174/1871527313666140917095514 25230234 PMC5193501

[B2] Athauda D. Foltynie T. (2016). Insulin Resistance and Parkinson's Disease: A New Target for Disease Modification. Prog. Neurobiol. 145–146, 98–120. 10.1016/j.pneurobio.2016.10.001 27713036

[B3] Bharadwaj P. Wijesekara N. Liyanapathirana M. Newsholme P. Ittner L. Fraser P. (2017). The Link between Type 2 Diabetes and Neurodegeneration: Roles for Amyloid-β, Amylin, and Tau Proteins. J. Alzheimers Dis. 59, 421–432. 10.3233/JAD-161192 28269785

[B4] Bjorbaek C. Kahn B. B. (2004). Leptin Signaling in the central Nervous System and the Periphery. Recent Prog. Horm. Res. 59, 305–331. 10.1210/rp.59.1.305 14749508

[B5] Blalock E. M. Geddes J. W. Chen K. C. Porter N. M. Markesbery W. R. Landfield P. W. (2004). Incipient Alzheimer's Disease: Microarray Correlation Analyses Reveal Major Transcriptional and Tumor Suppressor Responses. Proc. Natl. Acad. Sci. U S A. 101, 2173–2178. 10.1073/pnas.0308512100 14769913 PMC357071

[B6] Chatterjee S. Peters S. A. Woodward M. Mejia Arango S. Batty G. D. Beckett N. (2016). Type 2 Diabetes as a Risk Factor for Dementia in Women Compared with Men: A Pooled Analysis of 2.3 Million People Comprising More Than 100,000 Cases of Dementia. Diabetes care 39, 300–307. 10.2337/dc15-1588 26681727 PMC4722942

[B7] Cheng H. Gang X. Liu Y. Wang G. Zhao X. Wang G. (2019). Mitochondrial Dysfunction Plays a Key Role in the Development of Neurodegenerative Diseases in Diabetes. Am. J. Physiol. Endocrinol. Metab. 318 (5), E750–E764. 10.1152/ajpendo.00179.02019 31714795

[B8] Ci L. Y. Liu D. S. Yang J. Q. Liu Y. Z. Li C. L. Zhang X. (2018). Expression of Long Non-coding RNA and mRNA in the hippocampus of M-ice with T-ype 2 D-iabetes. Mol. Med. Rep. 18, 4960–4968. 10.3892/mmr.2018.9504 30272307 PMC6236254

[B9] Craft S. Watson G. S. (2004). Insulin and Neurodegenerative Disease: Shared and Specific Mechanisms. Lancet Neurol. 3, 169–178. 10.1016/S1474-4422(04)00681-7 14980532

[B10] Garcia-Garcia V. A. Alameda J. P. Page A. Casanova M. L. (2021). Role of NF-kappaB in Ageing and Age-Related Diseases: Lessons from Genetically Modified Mouse Models. Cells 10 (8), 1906. 10.3390/cells10081906 34440675 PMC8394846

[B11] Heidbreder M. Fröhlich F. Jöhren O. Dendorfer A. Qadri F. Dominiak P. (2003). Hypoxia Rapidly Activates HIF-3alpha mRNA Expression. FASEB J. 17, 1541–1543. 10.1096/fj.02-0963fje 12824304

[B12] Ho K. H. Yang X. Osipovich A. B. Cabrera O. Hayashi M. L. Magnuson M. A. (2020). Glucose Regulates Microtubule Disassembly and the Dose of Insulin Secretion via Tau Phosphorylation. Diabetes 69, 1936–1947. 10.2337/db19-1186 32540877 PMC7458041

[B13] Homma T. Fujii J. (2020). Emerging Connections between Oxidative Stress, Defective Proteolysis, and Metabolic Diseases. Free Radic. Res. 54, 931–946. 10.1080/10715762.2020.1734588 32308060

[B14] Iglesias-Gato D. Wikström P. Tyanova S. Lavallee C. Thysell E. Carlsson J. (2016). The Proteome of Primary Prostate Cancer. Eur. Urol. 69, 942–952. 10.1016/j.eururo.2015.10.053 26651926

[B15] Jadiya P. Kolmetzky D. W. Tomar D. Di Meco A. Lombardi A. A. Lambert J. P. (2019). Impaired Mitochondrial Calcium Efflux Contributes to Disease Progression in Models of Alzheimer's Disease. Nat. Commun. 10, 3885. 10.1038/s41467-019-11813-6 31467276 PMC6715724

[B16] Kasai S. Richardson M. J. E. Torii S. Yasumoto K. I. Shima H. Igarashi K. (2017). Increase in Proapoptotic Activity of Inhibitory PAS Domain Protein via Phosphorylation by MK2. Febs J. 284, 4115–4127. 10.1111/febs.14300 29054108

[B17] Khacho M. Harris R. Slack R. S. (2019). Mitochondria as central Regulators of Neural Stem Cell Fate and Cognitive Function. Nat. Rev. Neurosci. 20, 34–48. 10.1038/s41583-018-0091-3 30464208

[B18] Kraeuter A. K. Guest P. C. Sarnyai Z. (2019). The Y-Maze for Assessment of Spatial Working and Reference Memory in Mice. Methods Mol. Biol. 1916, 105–111. 10.1007/978-1-4939-8994-2_10 30535688

[B19] Kuhad A. Bishnoi M. Tiwari V. Chopra K. (2009). Suppression of NF-Kappabeta Signaling Pathway by Tocotrienol Can Prevent Diabetes Associated Cognitive Deficits. Pharmacol. Biochem. Behav. 92, 251–259. 10.1016/j.pbb.2008.12.012 19138703

[B20] Kulic L. Wollmer M. A. Rhein V. Pagani L. Kuehnle K. Cattepoel S. (2011). Combined Expression of Tau and the Harlequin Mouse Mutation Leads to Increased Mitochondrial Dysfunction, Tau Pathology and Neurodegeneration. Neurobiol. Aging 32, 1827–1838. 10.1016/j.neurobiolaging.2009.10.014 19942317

[B21] Li J. M. Zhao Y. Sun Y. Kong L. D. (2020). Potential Effect of Herbal Antidepressants on Cognitive Deficit: Pharmacological Activity and Possible Molecular Mechanism. J. Ethnopharmacol 257, 112830. 10.1016/j.jep.2020.112830 32259666

[B22] Lu S. Zhang H. Wei X. Huang X. Chen L. Jiang L. (2019). 2-dodecyl-6-methoxycyclohexa-2,5-diene-1,4-dione Isolated from *Averrhoa carambola* L. Root Ameliorates Diabetic Nephropathy by Inhibiting the TLR4/MyD88/NF-Κb Pathway. Diabetes Metab. Syndr. Obes. 12, 1355–1363. 10.2147/DMSO.S209436 31496773 PMC6689538

[B23] Lueptow L. M. (2017). Novel Object Recognition Test for the Investigation of Learning and Memory in Mice. J. visualized experiments : JoVE 126, 55718. 10.3791/55718 PMC5614391 28892027

[B24] Main A. M. Gillberg L. Jacobsen A. L. Nilsson E. Gjesing A. P. Hansen T. (2016). DNA Methylation and Gene Expression of HIF3A: Cross-Tissue Validation and Associations with BMI and Insulin Resistance. Clin. Epigenetics 8, 89. 10.1186/s13148-016-0258-6 27594926 PMC5010678

[B25] Makino Y. Kanopka A. Wilson W. J. Tanaka H. Poellinger L. (2002). Inhibitory PAS Domain Protein (IPAS) is a Hypoxia-Inducible Splicing Variant of the Hypoxia-Inducible Factor-3alpha Locus. J. Biol. Chem. 277, 32405–32408. 10.1074/jbc.C200328200 12119283

[B26] Moran C. Beare R. Phan T. G. Bruce D. G. Callisaya M. L. Srikanth V. (2015). Type 2 Diabetes Mellitus and Biomarkers of Neurodegeneration. Neurology 85, 1123–1130. 10.1212/WNL.0000000000001982 26333802 PMC5573049

[B27] Nguyen T. T. Ta Q. T. H. Nguyen T. T. D. Le T. T. Vo V. G. (2020). Role of Insulin Resistance in the Alzheimer’s Disease Progression. Neurochem. Res. 45, 1481–1491. 10.1007/s11064-020-03031-0 32314178

[B28] Ola M. S. Nawaz M. Ahsan H. (2011). Role of Bcl-2 Family Proteins and Caspases in the Regulation of Apoptosis. Mol. Cel Biochem 351, 41–58. 10.1007/s11010-010-0709-x 21210296

[B29] Ordonez D. G. Lee M. K. Feany M. B. (2018). α-Synuclein Induces Mitochondrial Dysfunction through Spectrin and the Actin Cytoskeleton. Neuron 97, 108–124.e6. 10.1016/j.neuron.2017.11.036 29249285 PMC5755717

[B30] Pagano G. Polychronis S. Wilson H. Giordano B. Ferrara N. Niccolini F. (2018). Diabetes Mellitus and Parkinson Disease. Neurology 90, e1654–e1662. 10.1212/WNL.0000000000005475 29626177

[B31] Pfeiffer S. Krüger J. Maierhofer A. Böttcher Y. Klöting N. El Hajj N. (2016). Hypoxia-inducible Factor 3A Gene Expression and Methylation in Adipose Tissue is Related to Adipose Tissue Dysfunction. Sci. Rep. 6, 27969. 10.1038/srep27969 27346320 PMC4921806

[B32] Platt T. L. Beckett T. L. Kohler K. Niedowicz D. M. Murphy M. P. (2016). Obesity, Diabetes, and Leptin Resistance Promote Tau Pathology in a Mouse Model of Disease. Neuroscience 315, 162–174. 10.1016/j.neuroscience.2015.12.011 26701291 PMC4715963

[B33] Pradelli L. A. Bénéteau M. Ricci J. E. (2010). Mitochondrial Control of Caspase-dependent and -independent Cell Death. Cell Mol Life Sci 67, 1589–1597. 10.1007/s00018-010-0285-y 20151314 PMC11115767

[B34] Quintanilla R. A. Matthews-Roberson T. A. Dolan P. J. Johnson G. V. (2009). Caspase-cleaved Tau Expression Induces Mitochondrial Dysfunction in Immortalized Cortical Neurons: Implications for the Pathogenesis of Alzheimer Disease. J. Biol. Chem. 284, 18754–18766. 10.1074/jbc.M808908200 19389700 PMC2707209

[B35] Santiago J. A. Bottero V. Potashkin J. A. (2019). Transcriptomic and Network Analysis Highlight the Association of Diabetes at Different Stages of Alzheimer’s Disease. Front. Neurosci. 13, 1273. 10.3389/fnins.2019.01273 31849586 PMC6895844

[B36] Sato N. Morishita R. (2014). Brain Alterations and Clinical Symptoms of Dementia in Diabetes: Aβ/tau-dependent and Independent Mechanisms. Front. Endocrinol. 5, 143. 10.3389/fendo.2014.00143 PMC4155814 25250014

[B37] Scherzer C. R. Grass J. A. Liao Z. Pepivani I. Zheng B. Eklund A. C. (2008). GATA Transcription Factors Directly Regulate the Parkinson's Disease-Linked Gene Alpha-Synuclein. Proc. Natl. Acad. Sci. U.S.A. 105, 10907–10912. 10.1073/pnas.0802437105 18669654 PMC2504800

[B38] Sheng Z. H. Cai Q. (2012). Mitochondrial Transport in Neurons: Impact on Synaptic Homeostasis and Neurodegeneration. Nat. Rev. Neurosci. 13, 77–93. 10.1038/nrn3156 22218207 PMC4962561

[B39] Sims-Robinson C. Kim B. Rosko A. Feldman E. L. (2010). How Does Diabetes Accelerate Alzheimer Disease Pathology. Nat. Rev. Neurol. 6, 551–559. 10.1038/nrneurol.2010.130 20842183 PMC3199576

[B40] Sterling T. Irwin J. J. (2015). ZINC 15--Ligand Discovery for Everyone. J. Chem. Inf. Model. 55, 2324–2337. 10.1021/acs.jcim.5b00559 26479676 PMC4658288

[B41] Stoothoff W. Jones P. B. Spires-Jones T. L. Joyner D. Chhabra E. Bercury K. (2009). Differential Effect of Three-Repeat and Four-Repeat Tau on Mitochondrial Axonal Transport. J. Neurochem. 111, 417–427. 10.1111/j.1471-4159.2009.06316.x 19686388 PMC2831412

[B42] Torii S. Goto Y. Ishizawa T. Hoshi H. Goryo K. Yasumoto K. (2011). Pro-apoptotic Activity of Inhibitory PAS Domain Protein (IPAS), a Negative Regulator of HIF-1, through Binding to Pro-survival Bcl-2 Family Proteins. Cell Death Differ 18, 1711–1725. 10.1038/cdd.2011.47 21546903 PMC3190112

[B43] Torii S. Kasai S. Suzuki A. Todoroki Y. Yokozawa K. Yasumoto K. I. (2015). Involvement of Inhibitory PAS Domain Protein in Neuronal Cell Death in Parkinson’s Disease. Cell Death Discov 1, 15015. 10.1038/cddiscovery.2015.15 27551449 PMC4981001

[B44] Wei X. Xu X. Chen Z. Liang T. Wen Q. Qin N. (2018). Protective Effects of 2-Dodecyl-6-Methoxycyclohexa-2,5 -Diene-1,4-Dione Isolated from *Averrhoa carambola* L. (Oxalidaceae) Roots on Neuron Apoptosis and Memory Deficits in Alzheimer’s Disease. Cell Physiol Biochem 49, 1064–1073. 10.1159/000493289 30196278

[B45] Yadav A. Kataria M. A. Saini V. Yadav A. (2013). Role of Leptin and Adiponectin in Insulin Resistance. Clin. Chim. Acta 417, 80–84. 10.1016/j.cca.2012.12.007 23266767

[B46] Yaffe K. Blackwell T. Kanaya A. M. Davidowitz N. Barrett-Connor E. Krueger K. (2004). Diabetes, Impaired Fasting Glucose, and Development of Cognitive Impairment in Older Women. Neurology 63, 658–663. 10.1212/01.wnl.0000134666.64593.ba 15326238

[B47] Yermakov L. M. Griggs R. B. Drouet D. E. Sugimoto C. Williams M. T. Vorhees C. V. (2019). Impairment of Cognitive Flexibility in Type 2 Diabetic Db/db Mice. Behav. Brain Res. 371, 111978. 10.1016/j.bbr.2019.111978 31141724 PMC6579681

[B48] Zhang Y. Chen Y. Qu H. Wang Y. (2019). Methylation of HIF3A Promoter CpG Islands Contributes to Insulin Resistance in Gestational Diabetes Mellitus. Mol. Genet. Genomic Med. 7, e00583. 10.1002/mgg3.583 30743315 PMC6465726

[B49] Zhang Y. Huang N. Q. Yan F. Jin H. Zhou S. Y. Shi J. S. (2018). Diabetes Mellitus and Alzheimer's Disease: GSK-3β as a Potential Link. Behav. Brain Res. 339, 57–65. 10.1016/j.bbr.2017.11.015 29158110

[B50] Zheng N. Lin X. Wen Q. KintokoZhang S. Zhang S. Huang J. (2013). Effect of 2-Dodecyl-6-Methoxycyclohexa-2,5-Diene-1,4-Dione, Isolated from *Averrhoa carambola* L. (Oxalidaceae) Roots, on Advanced Glycation End-Product-Mediated Renal Injury in Type 2 Diabetic KKAy Mice. Toxicol. Lett. 219, 77–84. 10.1016/j.toxlet.2013.03.001 23500658

[B51] Zilliox L. A. Chadrasekaran K. Kwan J. Y. Russell J. W. (2016). Diabetes and Cognitive Impairment. Curr. Diab Rep. 16, 87. 10.1007/s11892-016-0775-x 27491830 PMC5528145

